# Contraceptive use in women with inherited metabolic disorders: a retrospective study and literature review

**DOI:** 10.1186/s13023-022-02188-x

**Published:** 2022-02-08

**Authors:** Jessica I. Gold, Nina B. Gold, Diva D. DeLeon, Rebecca Ganetzky

**Affiliations:** 1 Division of Human Genetics, Section of Biochemical Genetics, The Children’s Hospital of Philadelphia, 3401 Civic Center Blvd, Philadelphia, PA 19104 USA; 2 Division of Medical Genetics and Metabolism, Massachusetts General Hospital for Children, Boston, MA USA; 3 Division of Endocrinology and Diabetes, The Children’s Hospital of Philadelphia, Philadelphia, PA USA; 4 Department of Pediatrics, Perelman School of Medicine at the University of Pennsylvania, Philadelphia, PA USA; 5 Division of Human Genetics, Section of Biochemical Genetics, The Children’s Hospital of Philadelphia, Philadelphia, PA USA

**Keywords:** Contraception, Reproductive planning, Adult metabolic medicine, Inherited metabolic disorders, Pregnancy, Birth control

## Abstract

**Background:**

Reproductive planning is an emerging concern for women with inherited metabolic disease (IMD). Anticipatory guidance on contraception is necessary to prevent unintended pregnancies in this population. Few resources exist to aid informed decision-making on contraceptive choice. A retrospective case–control study was performed to examine trends in reproductive planning for adolescent and adult women seen at the Children’s Hospital of Philadelphia (CHOP). Literature review on contraception and IMD was performed to assess global use.

**Results:**

In a cohort of 221 reproductive-aged female IMD patients, 29.4% reported routine contraceptive use. Anticipatory guidance on contraception was provided by metabolic physicians to 36.8% of patients during the study period. Contraception discussion was more likely to occur in women older than 21 years, who lived independently and were followed by gynecology. Women who received contraception counseling from their metabolic physician were 40-fold more likely to use regular contraception. Use of combined hormonal contraceptives was most commonly reported, but contraception choice varied by age and IMD.

**Conclusion:**

Metabolic physicians are ideally suited to provide guidance on contraception to women with IMD. Reproductive planning should be addressed routinely using shared decision-making. Contraceptives should be selected for their efficacy, effects on metabolism, and likelihood of patient adherence.

## Background

Due to expanded newborn screening and medical advancements in care and diagnosis, people with inherited metabolic disorders (IMD) are surviving into adulthood with improved health. As these individuals age, there is a growing need for counseling on reproductive care [1, 2]. For many women with IMD, pregnancy carries risks both to the patient and to the developing fetus, particularly if the pregnancy is unintended and the underlying IMD is inadequately managed. Some hormonal contraceptives may disrupt metabolism, leading to adverse outcomes. As such, counseling on contraceptive selection is important for women with IMD. Because patients with IMD often have long-lasting and frequent contact with their metabolic physicians, these clinicians are well-positioned to facilitate anticipatory guidance discussions on reproductive care.

The World Health Organization (WHO) has published guidelines for contraception in the setting of common medical disorders, including liver disease, hypertension, and diabetes. No centralized information on best practices for women with rare diseases, including IMD, exists. Contraceptives vary widely in their efficacy, mechanism of action, usage, and side effects. For women with medical comorbidities, it is critical to balance the risks of any contraceptive method with the adverse outcome of an unintended pregnancy [3, 4]. This study and review of the literature aims to examine trends in reproductive planning for women with IMD at one center and propose considerations for counseling on contraceptive selection.

## Results

The Metabolism section at CHOP followed 221 reproductive-aged females, including 111 adolescents 12–21 years old, and 110 adults 22–50 years old between January 2012 and December 2019. Most adolescent patients live with a parent or caregiver (93%), while half of the adult patients live independently (50.9%). Intellectual disability (ID) has been diagnosed in 37.1% of our cohort, affecting 41.4% of adolescents and 32.7% of adults.

**Fig. 1:**
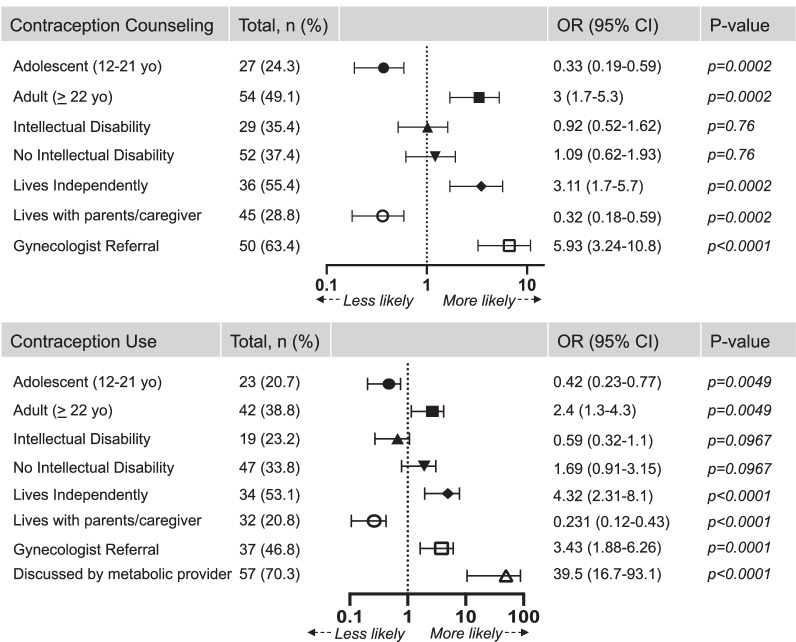
Counseling on contraception was significantly increased in adult patients, patients who live independently and patients who see a gynecologist. Contraceptive use was significantly increased in adult patients, patients who live independently, patients who see a gynecologist, and patients who discussed contraception with their metabolic physician. Odds ratio (OR), 95% confidence interval (CI), and p-value displayed for all categories

A metabolic physician documented counseling about contraception use during at least one visit with 36.8% of reproductive-aged patients. Discussions were significantly more likely to occur with patients who were adults (OR 3.3, CI 1.9–5.9), live independently (OR 3.1, CI 1.7–5.7), or are followed by gynecologists (OR 5.93, CI 3.24–10.8) (Fig. 1). The presence of ID did not significantly affect introduction of this topic. Routine contraceptive use was reported by 29.4% of patients, including 20.7% of adolescents and 38.9% of adults (Fig. 1). Contraception use was significantly higher in adult patients (OR 2.6, CI 1.4–4.6), patients living independently (OR 4.23, CI 2.3–7.9), and patients seeing gynecologists (OR 3.43, CI 1.88–6.26). Most notably, contraceptive use was 40-fold more likely in patients who received anticipatory guidance from their metabolic provider. Counseling on contraception by a metabolic physician led to initiation of use within 2.37 ± 1.3 visits.

**Fig. 2:**
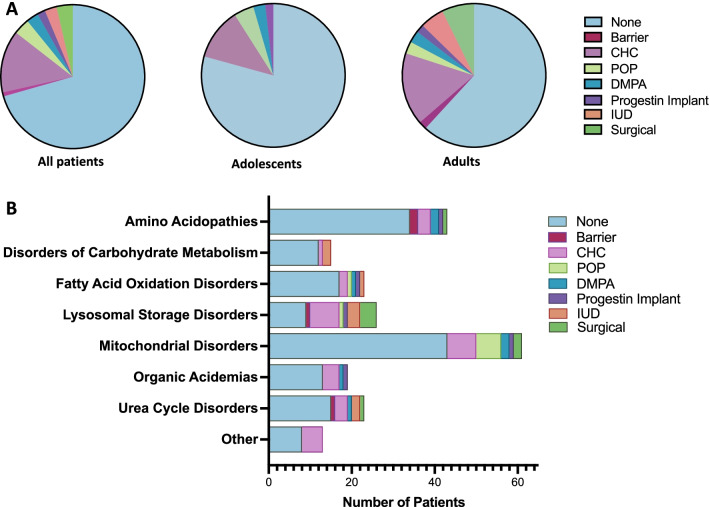
Contraceptive method varies by age and IMD. **A** Current documented contraceptive method in all reproductive-aged women followed by the CHOP metabolism clinic (n = 221, *left*), adolescent women (n = 111, *middle*), and adult (n = 110, *right*). **B** Current and historical contraception use according to IMD diagnosis. *CHC* combined hormonal contraception; *POP* progestin-only pills; *IUD* intrauterine device

Combined hormonal contraceptives (CHC), including the pill, patch, and vaginal ring were most commonly used (Fig. 2A). Long-acting reversible contraception (LARC), including reversible forms (IUD or progesterone implant) and surgical methods, was more likely to be used by adults compared to adolescents (OR 6.46, CI 1.33–31.3). We also examined use of contraception according to underlying metabolic diagnosis (Fig. 2B). Current and historic forms of contraception were included. Contraception use varied among IMD class. Patients with lysosomal storage disorders, specifically Gaucher disease, were most likely to use contraception, with the majority opting for CHCs or surgical methods. Of note, no patient in our cohort with homocystinuria or a hepatic glycogen storage disorder (GSD) had ever used a CHC, which is contraindicated in these disorders [5–7].

Many patients at high risk for adverse events during pregnancy were not using contraception. Most patients with urea cycle disorders (UCD) and organic acidemias did not report contraception use (65% and 68% respectively). Among patients with phenylketonuria (PKU), 77% had no contraceptive use ever documented, despite consensus recommendations [8]. Anticipatory guidance regarding pregnancy and PKU was not universally provided. Discussion on the risks of uncontrolled PKU in pregnancy only occurred for 30% of adolescent patients and 66.7% of adult patients. Only 25% of patients on pegvaliase and 40% of patients on eliglustat reported contraceptive use, despite it being a prerequisite for these medications.

### Literature review

Review of literature on IMD and contraception primarily revealed case reports. There is no comprehensive resource on contraceptive use, efficacy, or adverse events in IMD. Guidelines on homocystinuria, GSD I and III, and Wilson disease provided the most specific recommendations. Estrogen-containing contraception is contraindicated in homocystinuria due to the risk of thrombosis and stroke [9]. Estrogen-containing contraceptives are also discouraged in hepatic GSD due to their connection to the formation and growth of hepatic adenomas [7, 10, 11]. Ethinyl estradiol may increase triglycerides, a concern in hepatic GSD, and familial hyperlipidemias [7, 11, 12]. Copper-containing IUDs are discouraged in Wilson disease due to theoretical copper absorption. International guidelines on PKU encouraged contraceptive use without providing a preferred methods [8, 13–15]. We compiled contraception recommendations for specific IMDs based on the literature reviewed (Table 1).

**Table 1:** Studies addressing contraceptive use in IMD

Disorder	Recommendation	References
Cystathionine β-synthase deficiency	Avoid estrogen-containing contraception due to increased risk of thrombosis	Morris et al. [5]
Galactosemia	Counsel about adequate birth control methods as hormonal methods of cycle control may fail to prevent pregnancy in women with elevated FSH. IUD may provide the lowest failure rate	Welling et al. [34]
	Counsel patients that they have reduced fertility, not complete infertility	van Erven et al. [34]
	All oral contraceptive pills (OCP and POP) contain lactose as binding agents, recommend alternative methods	
Gaucher Disease	No known contraindications for CHC or progesterone-only contraception unless there is severe liver involvement	Granovsky-Grisaru et al. [35], Granovsky-Grisaru et al. [36]
	Avoid copper IUDs if patient is at risk for menorrhagia	Granovsky-Grisaru et al. [36]
	Contraception must be used by both men and women who are receiving Miglustat	Cox et al. [37]
Glycogen Storage Disease I	Avoid ethinylestradiol due to the link with hepatic adenomas. Recommend POP	Mairovitz et al. [6], Sechi et al. [7]
	Progestin-only contraceptives may have risks of reduced bone mineral density	Kishani et al. [11]
Glycogen Storage Disease Ib	Avoid use of IUDs due to potential risk of increased infection	Kishani et al. [11]
Glycogen Storage Disease III	Avoid estrogen-containing contraception due to risk of hepatic adenomas. If using progesterone-only contraceptive, monitor for reduced bone mineral density	Kishani et al. [38]
Familial Hypercholesterolemia	Recommend low estrogen-containing oral contraceptives, IUD, and barrier techniques. For women older than 35 years, IUDs and barrier techniques are preferred	Balla et al. [12], Watts et al. [39]
	Counsel on contraception prior to starting statin with reinforcement provided annually	Balla et al. [12], Watts et al. [39]
Hereditary Hemochromatosis	Use shared decision-making if menstrual suppression is indicated due to potential risk of elevated ferritin and need for phlebotomy	Kalinowski et al. [40]
Methylmalonic Acidemia/Propionic Acidemia	There are no known contraindications for the use of hormonal contraception. Discuss contraception and sexual health during adolescence	Baumgartner et al. [14]
Niemann Pick C	Contraception must be used by both men and women who are receiving Miglustat	Wraith et al. [41]
Phenylketonuria	Recommend the most effective form of contraceptive	van Wegberg et al. [8], AAP [15]
	Prior to conception, continue contraception until phenylalanine levels are within target range for at least 2 weeks	van Wegberg et al. [8], van Spronsen et al. [42]
	Begin age-related sexual education and guidance on risk for maternal PKU syndrome at age 12	van Wegberg et al. [8], AAP [15], Camp et al. [43]
	Develop a robust transition program so that young adult women are not lost to follow-up	van Wegberg et al. [8]
Wilson’s disease	Avoid estrogen-containing contraception and copper IUD. Recommend progesterone-only contraception, barrier methods, and spermicides	Connolly et al. [44], Haimov-Kochman et al. [45]; EASL [46], Kathawala [47], Patil et al. [48]

*CHC* combined hormonal contraception; *IUD* intrauterine device; *OCP* oral contraceptive pills; *PKU* phenylketonuria; *POP* progestin-only pills

## Discussion

Reproductive health planning is an emerging, crucial issue for people with IMDs. Contraception limits risks to patients with IMD and their offspring, as it allows for adequate reproductive planning and early pregnancy monitoring. Contraception is therefore a necessary component of the healthcare of adults and adolescents with IMD.

Contraception counseling was most likely to occur with adult women who live independently. These women are likely to attend visits independently or with a partner, avoiding the potential discomfort of addressing sexual health in front of parents or caregivers. Adult women living independently may demonstrate higher career focus making them more likely to use contraception to delay pregnancy [16]. Gynecology referral had a positive impact on contraceptive use. LARC have the lowest failure rate and were used significantly more frequently in females followed by gynecologists (with gynecology: 9/81; without gynecology: 4/121, p < 0.04). Joint management with gynecology allows for a greater range of contraceptive options.

Diagnosis of ID had an equivocal effect on both contraception counseling and use of contraception. This runs counter to current literature on sexual health in women with ID, where the presence of ID decreases the likelihood of reproductive planning [17]. Metabolic physicians may be uniquely suited to have such conversations with patients with ID because of strong rapport with patients and their families due to longstanding relationships. For our cohort with ID, there was no significant benefit of gynecology consultation on contraceptive use (OR 2.45, CI 0.86–6.96, p = 0.092). In contrast, metabolic physician-led counseling on contraception significantly improves actual usage among women with ID and IMD (OR 4.8, CI 1.46–15.8, p = 0.01) Most women with ID in our cohort opted for daily oral contraception, such as POP or OCP (73%), and only one patient chose a LARC (IUD). We speculate that these women are already taking several daily medications and, therefore, are not burdened by adding additional daily oral medications. It is also possible that there is insufficient awareness about LARC in this population or limited options for placement.

For all cohorts, metabolic physicians are well-poised to initiate conversations about reproductive health. In our clinic, engaging women even once about reproductive planning led to a 40-fold increase in contraceptive use (Fig. 1). Patients were more likely to initiate contraception on the advice of their metabolic physician compared to their gynecologist. In our clinical experience, patients with IMD frequently defer to their metabolic physician for advice on any new medication or supplement. This highlights the critical role that the metabolic physician plays in encouraging contraception.

Unfortunately, overall, contraceptives were used only by a minority of patients across all classes of IMD, increasing the risk for unintended pregnancy (Fig. 2). This was true even in conditions where pregnancy was associated with high risk for adverse pregnancy outcomes. Only 23% of women with PKU have ever used contraception, with slightly higher percentages for female patients with organic acidemias (32%) and UCDs (35%). This resulted in an unacceptably high rate of maternal PKU syndrome (1/12 pregnancies), which causes irreversible neurologic damage to the fetus. These data are similar to those found in other studies showing that reproductive health is often omitted from healthcare transition planning [18]. In general, women with chronic diseases are less likely to use contraception, despite the risks of unintended pregnancy [19, 20]. While IMD-related comorbidities may preclude reproduction for some, many women with IMD are sexually active and should be receive anticipatory guidance on contraception.

One barrier to universal metabolic counseling on contraception is the lack of systematic resources. In the IMD literature, most recommendations are provided on an ad hoc, case report basis. Contraception was addressed in 14.3% (12/84) of clinical practice guidelines (Table 2). The lack of expert opinion minimizes the importance of reproductive health counseling and fails to provide guidance for metabolic physicians. Misperceptions may exist about sexual activity in patients with IMD, especially those that are evaluated in a pediatric context or who still live with a caregiver, and providers may feel discomfort discussing this topic with patients whom they have known since childhood [21]. Counseling through educational hand-outs or inclusion of questionnaires in the electronic health record could lead to uniform guidance on contraception. Likewise, incorporation of healthcare contracts may encourage contraceptive use prior to starting potentially teratogenic medications, such as pegvaliase. Similar processes, for example the iPledge program for isoretinol use, have shown efficacy in preventing unintended pregnancy in analogous contexts [22].

**Table 2:** Sex hormone effects on metabolism

Hormone	Effect on metabolism	Example affected conditions	Author
Estrogen	Increases total cholesterol, triglycerides, and high-density lipoproteins, decreases low-density lipoproteins	Cholesterol biosynthesis disorders, Cholesterol storage disorders, GSD	Winkler et al. [48], Nash et al. [49], Ruoppolo et al. [50]
	Increases circulating glucose and insulin	FAOD, GSD, disorders with risk of hypoglycemia	Winkler et al. [48], Godsland et al. [51]
	Decreases muscle glucose uptake via repression of GLUT4 expression	Muscle-predominant GSD	Barros et al. [52]
	Decreased absorption of B vitamins, specifically riboflavin, thiamine, and pyridoxine	PDH deficiency, Homocystinuria	Anderson et al. [53], Webb et al. [54], Rose et al. [55]
	Increased risk of venous thromboembolism	Homocystinuria, Cobalaminopathies	Den Heijer et al. [23]
Progestins	Increase triglycerides and high-density lipoproteins, decreases low-density lipoprotein	Cholesterol biosynthesis disorders, Cholesterol storage disorders, GSD	Godsland et al. [51], Butler et al. [56]
	Decrease bone mineral density with DMPA	Disorders with risk for poor bone health	ACOG [4]
Combined estrogen and progestin	Changes in plasma amino acid profiles: decreases glutamine, glycine, proline, lysine, hydroxyproline, ornithine, tyrosine and increases isoleucine and phenylalanine	PKU, IVA, GAMT deficiency, Lysinuric Protein Intolerance	Ruoppolo et al. [50], Wang et al. [57]
	Increase total cholesterol, triglycerides, and high-density lipoproteins	Cholesterol biosynthesis disorders, Cholesterol storage disorders, GSD	Wang et al. [57]
	Decrease free and total carnitine	Carnitine Uptake deficiency, Secondary carnitine deficiency	Bach et al. [58]
	Increase cholic acid	Disorders of bile acid metabolism	Connolly et al. [44]
	Decrease chenodeoxycholic acid	Cerebrotendinous Xanthomatosis	Connolly et al. [44]
	Increase ceruloplasmin	Wilson disease	Roberts et al. [59]

*GSD* glycogen storage disease; *FAOD* fatty acid oxidation disorders; *PDH* pyruvate dehydrogenase; *PKU* phenylketonuria, *IVA* isovaleric acidemia; *GAMT* guanidinoacetate methyltransferase

This study is limited by the setting of our cohort. CHOP is a stand-alone children’s hospital. It is not affiliated with an adult hospital. The Section of Metabolic Disease at CHOP follows all-aged patients (currently newborn to 88 years-old). Adults with disorders of intermediary metabolism continue to be admitted to CHOP for metabolic crises and other emergent care. There are six attending physicians within the section, all of whom are trained in pediatrics and clinical genetics. Adults are frequently seen by their childhood metabolic physician. Potential barriers include lack of experience in discussing or providing contraception, limited knowledge on contraceptive options, scant training on offering adult-centered care, and discomfort with shared decision-making. Our cohort, with only 30% contraceptive use, may not be generalizable to metabolism clinics specifically dedicated to adult-centered care. Metabolic physicians with training in internal medicine, family medicine, or obstetrics-gynecology have a stronger background in discussing and prescribing contraception, leading to higher rates of compliance with contraceptives.

Another possibility is that reproductive healthcare is lacking throughout the practice of metabolism. Many IMD were previously thought to be life-limiting with accompanying decrease in reproductive fitness. The advent of newborn screen, novel therapeutics, and improved care created a new population of adults who require specialized management. Women with IMD require more uniformity in counseling on the adverse effects of unintended pregnancy and how contraception can ameliorate these risks. Metabolic physicians can serve as leaders in this area, working cooperatively with gynecology or primary care providers to best support patients’ decision-making. To empower metabolism-driven discussion of contraception, we summarize important aspects of counseling on contraception for women with IMD below.

### Contraceptive counseling for women with IMD

To provide optimal reproductive planning advice to women with IMD, both the efficacy of the method and the effects of sex hormones on metabolism should be considered. The WHO recommends using the contraceptive with the lowest failure rate in women with health risks due to unintended pregnancy (currently LARC, including the hormonal and non-hormonal IUD and the progestin implant) [3, 4]. Estrogen and progestin, both separately and combined as CHC are responsible for several changes to metabolism in healthy women (Table 2). Patients’ PCPs or gynecologists may not consider the side effects of contraception on metabolic pathways, placing the metabolic physician in a unique role to suggest the method most compatible with the patient’s diagnosis.

IMD patients’ comorbidities also play an important role in contraceptive choice. Use of CHC is contraindicated in women with a history of hypertension, cardiac disease, or stroke (21.5% of our patient cohort) [3, 4]. Migraines with aura, a common manifestation of mitochondrial disease and reported in 23.1% of our entire cohort, are another contraindication for CHC use due to the risk of stroke [12]. Women with a history or increased risk for venous thromboembolism due to homocystinuria, cobalaminopathies and other thrombophilic IMDs also should not receive CHC [23, 24]. Women with IMD may be the recipients of solid organ transplants, including liver and kidney. Most contraceptive methods are safe to use after uncomplicated organ transplant, but CHC are contraindicated in women with acute or chronic graft rejection [3, 25]. IUD have also been proven to be safe post-transplant, with no increased risk of developing pelvic infections [26]. Depot medroxyprogesterone (DMPA) and IUD are preferred for women on anti-epileptic drugs due to potential medication interactions (29.4% of our cohort) [3]. Partnering with gynceology may be beneficial for identifying the contraceptive with the safest side effect profile.

Contraception may improve IMD symptoms. The guidelines for propionic acidemia/methylmalonic acidemia recommend hormonal contraception for management of perimenstrual metabolic instability [14]. Several cases of catamenial hyperammonemia in UCDs have been described, which resolved with menstrual suppression using DMPA or CHC [27–29]. Hormonal contraception may also limit perimenstrual aggression in Smith–Lemli–Opitz Syndrome and catamenial porphyria crises in acute intermittent porphyria [30, 31]. In our cohort, 16 patients reported catamenial metabolic decompensations, of which 5 (31%) utilized hormonal contraception for management.

### Discussing reproductive health with IMD patients

It is important to address surgical methods of contraception or sterilization due to its history with the eugenics movement. The United Nations convention on the rights of persons with disabilities states that “people with disabilities must have access on an equal basis to all forms of sexual and reproductive health care [3].” For women with intellectual disability, supported decision-making should occur with a trusted caregiver who will act in the best interests of the patient. Decision-makers should consider social factors, protection against sexual abuse, and support options through potential pregnancy and parenthood [32]. They should also have valid grounds for desiring sterilization, such as avoidance of grave harm from pregnancy or inability to use a reversible contraceptive. For women with IMD, consideration of the metabolic risks of pregnancy and labor must also be weighed. The popularity and effectiveness of LARCs, including the IUD and the progestin implant, may make sterilization a rarer choice [33].

Finally, genetic counseling should occur at least once with adolescent and adult IMD patients. Counseling should address disease inheritance, recurrence risk, and options for genetic testing during antenatal and prenatal periods. Prospective parents should also be informed that variable expressivity occurs between generations for many IMD, making disease course less predictable in offspring. Additionally, discussions about reproductive health may lead to improved care coordination with patients’ primary care and OB providers. These providers may need education about risks during and after pregnancy related to IMDs. Pediatricians of patients’ future offspring should also be involved in care, as they may be directing the initial screening or management of newborn infants.

## Conclusion

Use of contraception to prevent unintended pregnancy and catamenial exacerbation of symptoms is important for women with IMD. In our cohort, contraception counseling by a metabolic physician is the strongest predictive factor for routine contraceptive use. Metabolic providers should utilize their trusted relationships with their patients to broach discussions about reproductive health, Advice on contraceptive choice should consider the contraceptive’s effects on metabolism, efficacy, comorbidities, side effects, and patient preference. CHC have the most effects on metabolism and may not be the optimal choice for women with IMD. LARC, such as IUD and progestin-implants, have not been studied extensively in this population, but are likely to be well-tolerated and should be considered first line. Greater collaboration with gynecology may encourage greater diversity in contraceptive options, especially LARC. Overall, the decision regarding contraceptive use is best made on an individual basis in concert between the adolescent or adult IMD patient and her physician.

## Patients and methods

### Patients

A retrospective chart review was conducted for all female patients with ages between 12–50 years-old followed by the Division of Genetics and Metabolism at Children’s Hospital of Philadelphia (CHOP) (n = 256). Patients with at least two visits with a clinical metabolic physician between January 2012 and December 2019 and a known diagnosis of an IMD (urea cycle disorder, fatty acid oxidation disorder, amino acidopathy, lysosomal storage disease, organic acidemia, mitochondrial disorder, glycogen storage disorder) were included. The two-visit criterion was used to ensure that only patients for whom CHOP was their established metabolic care center were included. We also excluded patients documented to be premenarchal or menopausal during the length of our study period (n = 35). Demographics of our study population meeting inclusion criteria can be found in Table 3.

**Table 3:** Demographics and clinical characteristics of reproductive-aged female patients meeting inclusion criteria

	Total	Adolescents (12–21)	Adults (> 22)
Number	221	111	110
Age (mean ± SD)	25.2 ± 11.3	16.2 ± 2.6	25.2 ± 9.3
Insurance (n, %)			
Private only	117 (52.9)	59 (53.2)	58 (52.7)
Medicare only	5 (2.3)	1 (0.9)	4 (3.6)
Medicaid only	39 (17.6)	22 (19.8)	17 (15.5)
Self pay only	1 (0.5)	0 (0)	1 (0.9)
Private + Medicare	4 (1.8)	1 (0.9)	3 (2.7)
Private + Medicaid	42 (19)	26 (23.4)	16 (14.5)
Medicaid + Medicare	12 (5.4)	2 (1.8)	10 (9)
Private + Medicaid + Medicare	1 (0.5)	0 (0)	1 (0.9)
Race (n, %)			
African American	25 (11.3)	12 (10.8)	13 (11.8)
Asian	5 (2.3)	3 (2.7)	2 (1.8)
Hispanic	14 (6.3)	9 (8.1)	5 (4.5)
White	155 (70.1)	69 (62.2)	86 (78.2)
Other	22 (10)	18 (16.2)	4 (3.6)
Residence (n, %)			
Lives with parents/caregiver	154 (69.7)	103 (92.8)	51 (46.3)
Lives independently	64 (29)	8 (7.2)	56 (50.9)
Lives in group home	3 (1.4)	0 (0)	3 (2.7)
Intellectual disability (n, %)	82 (37.1)	46 (41.4)	36 (32.7)
Diagnosis (n, %)			
Aminoacidopathy	41 (18.6)	19 (17.1)	22 (20)
Disorder of carbohydrate metabolism	15 (6.8)	11 (9.9)	4 (3.6)
Fatty acid oxidation disorder	21 (9.5)	15 (13.5)	6 (5.5)
Lysosomal storage disorder	31 (14)	9 (8.1)	22 (20)
Mitochondrial disorder	59 (26.7)	28 (25.2)	31 (28.2)
Organic acidemia	22 (10)	14 (12.6)	8 (7.2)
Urea cycle disorder	22 (10)	7 (6.3)	15 (13.6)
Other	10 (4.5)	8 (7.2)	2 (1.8)

### Statistical analysis

Logistic regression was used to identify factors associated contraceptive counseling and use. Results were reported as odds ratios (OR) with 95% confidence intervals. All analyses were conducted using Stata 16.1.

### Literature review

We queried the PubMed and Cochrane Library databases using the following search terms: “inborn error of metabolism & contraception,” “inborn error of metabolism & contraceptives,” “inborn error of metabolism & birth control” “inborn error of metabolism & estrogen” “inborn error of metabolism & progesterone” “inherited metabolic disorder & contraception,” “inherited metabolic disorder & contraceptives,” “inherited metabolic disease & birth control” “inherited metabolic disorder & estrogen,” and “inherited metabolic disorder & progesterone.” This search identified 31 primary articles. We also examined 84 international clinical practice guidelines on IMD for discussion of contraception.

## Data Availability

The datasets analyzed during the current study are available from the corresponding author upon request.

## Abbreviations

IMD: Inherited metabolic disorders
WHO: World Health Organization
CHOP: Children’s Hospital of Philadelphia
ID: Intellectual disability
OR: Odds ratio
CI: Confidence interval
CHC: Combined hormonal contraception
LARC: Long-acting reversible contraception
UCD: Urea cycle disorders
PKU: Phenylketonuria
LSD: Lysosomal storage disorder
DMPA: Depot medroxyprogesterone
IUD: Intrauterine device
OCP: Oral contraception pills
POP: Progestin-only pill
BMD: Bone mineral density
POI: Premature ovarian insufficiency
GSD: Glycogen storage disease
FAOD: Fatty acid oxidation disorder
PDH: Pyruvate dehydrogenase
GAMT: Guanidinoacetate methyltransferase
